# Efficacy and safety of ixekizumab in patients with active psoriatic arthritis with and without concomitant conventional disease-modifying antirheumatic drugs: SPIRIT-P1 and SPIRIT-P2 3-year results

**DOI:** 10.1007/s10067-022-06218-8

**Published:** 2022-06-08

**Authors:** Laura C. Coates, Philip Mease, Andris Kronbergs, Cameron Helt, David Sandoval, So Young Park, Bernard Combe, Peter Nash, Atul Deodhar

**Affiliations:** 1 Nuffield Department of Orthopaedics, Rheumatology and Musculoskeletal Sciences, University of Oxford, Oxford, UK; 2 Swedish Medical Center/Providence St, Joseph Health and University of Washington, Seattle, WA USA; 3 Eli Lilly and Company, Indianapolis, IN USA; 4 University of Montpellier, Montpellier, France; 5 Department of Medicine, University of Queensland, Queensland Brisbane, Australia; 6 Division of Arthritis and Rheumatic Diseases, Oregon Health & Science University, 3181 S.W. Sam Jackson Park Rd, Portland, OR 97239 USA

**Keywords:** Antirheumatic agents, Ixekizumab, Methotrexate, Psoriatic arthritis

## Abstract

**Introduction/objectives:**

To evaluate the three-year efficacy and safety of ixekizumab with and without concomitant conventional synthetic disease-modifying antirheumatic drug (csDMARD) use in patients with active psoriatic arthritis (PsA).

**Method:**

Patients with PsA who were biologic-naïve (SPIRIT-P1, NCT01695239) or had prior inadequate response to tumor necrosis factor inhibitors (SPIRIT-P2, NCT02349295) were randomized to receive 80-mg ixekizumab every four weeks after receiving 160-mg ixekizumab at baseline. Efficacy, safety, and immunogenicity were evaluated in this post-hoc analysis in three subgroups: (1) ixekizumab monotherapy, (2) ixekizumab and methotrexate (MTX), (3) ixekizumab and any csDMARD (including MTX). Missing data were imputed using multiple imputation for continuous variables and modified non-responder imputation for categorical variables.

**Results:**

Efficacy was similar across the three subgroups with 59.1%, 67.0%, and 66.1% of ixekizumab-treated patients achieving 20% improvement in the American College of Rheumatology scale score at week 156. Radiographic progression of structural joint damage (SPIRIT-P1 only) was similarly inhibited across the three subgroups with several outliers. No new safety signals were reported, and 91.0%, 84.1%, and 83.2% in the three subgroups reported ≥ 1 treatment-emergent adverse event. At week 156, 15.9%, 13.1%, and 11.0% in the three subgroups had antidrug antibodies; most had low titer status.

**Conclusions:**

Ixekizumab showed sustained efficacy in treating patients with PsA for up to three years in monotherapy or in combination with MTX or any csDMARD. The three subgroups had similar safety and immunogenicity profiles, which supports that the use of concomitant MTX or csDMARDs does not seem to impact the benefit/risk profile of ixekizumab.

**Table:** 

**Key Points** *• Ixekizumab treatment led to improved clinical responses over time when used as monotherapy or in combination with concomitant MTX or any concomitant csDMARD (including MTX) in patients with active PsA.* *• Ixekizumab monotherapy has similar radiographic efficacy as ixekizumab with MTX or ixekizumab with other csDMARDs (including MTX); similar inhibition of radiographic progression was observed between the subgroups of patients receiving ixekizumab monotherapy or ixekizumab with MTX or other csDMARDs.* *• The long-term safety profile of ixekizumab used as monotherapy or in combination with MTX or any other csDMARDs is consistent with what has been previously reported. The addition of MTX or any csDMARD to ixekizumab treatment did not negatively impact the favorable long-term safety profile of ixekizumab.*

**Supplementary Information:**

The online version contains supplementary material available at 10.1007/s10067-022-06218-8.

## Introduction

Psoriatic arthritis (PsA) is a chronic inflammatory, progressive, destructive disease that results in deformities, impaired physical function, and decreased quality of life [1]. Biologic disease-modifying antirheumatic drugs (bDMARDs) have demonstrated efficacy in treating patients with PsA. bDMARDs are often prescribed in combination with conventional synthetic disease-modifying antirheumatic drugs (csDMARDs); however, there is little evidence to support guidance on when to use biologic monotherapy versus concomitant treatment with csDMARDs. Although no differences in efficacy have been observed between patients treated with biologic drugs with or without methotrexate (MTX), specifically, or any concomitant csDMARD in randomized controlled trials [2–5], registry studies have shown that long-term differences in effectiveness via drug survival may be observed with tumor necrosis factor inhibitors [6–9]. The Group for Research and Assessment of Psoriasis and Psoriatic Arthritis (GRAPPA) 2015 Treatment Recommendations for Psoriatic Arthritis notes limited data available on combining therapies in PsA and that the use of concomitant MTX with bDMARDs does not appear to improve clinical symptoms beyond bDMARD monotherapy; however, results from registry studies have demonstrated greater drug survival when certain bDMARDs, particularly infliximab, are used with concomitant MTX [10]. The 2018 American College of Rheumatology(ACR)/National Psoriasis Foundation Guideline for the Treatment of Psoriatic Arthritis recommends bDMARD monotherapy over the use of concomitant MTX, noting that concomitant MTX may be advisable if the patient has severe psoriasis, a partial response to current MTX, or uveitis [11]. Concomitant MTX may also be suitable for patients receiving TNFi, particularly infliximab and adalimumab, to lessen immunogenicity [11]. The 2019 European League Against Rheumatism (EULAR) guideline’s stance on concomitant MTX aligns with the above recommendations from ACR [11]. Despite limited and conflicting evidence, MTX is the most common first-line treatment for PsA [12].

Ixekizumab, a specific inhibitor of the IL-17A cytokine, is approved in adults for the treatment of active PsA [13, 14]. In SPIRIT-P1 and SPIRIT-P2 24-week studies, ixekizumab demonstrated efficacy both as monotherapy and with background concomitant csDMARDs [9, 15]. Similar results were observed in a 52-week SPIRIT-P1 and SPIRIT-P2 study when ixekizumab was used as monotherapy or when added to concomitant MTX [16]. In a head-to-head study of ixekizumab versus adalimumab, ixekizumab had consistent efficacy regardless of concomitant MTX while ADA efficacy numerically increased with concomitant MTX [17]. The aim of this integrated analysis was to evaluate the long-term clinical efficacy, inhibition of radiographically assessed progression of structural damage, safety, and immunogenicity of ixekizumab through three years (156 weeks) in patients with active PsA enrolled in SPIRIT-P1 and SPIRIT-P2 according to concomitant csDMARD received in the following subgroups: (1) ixekizumab monotherapy (no concomitant MTX or other csDMARDs), (2) ixekizumab and MTX, (3) ixekizumab and any csDMARD (including MTX).

## Materials and methods

### Study design

This analysis included integrated data from the SPRIT-P1 (NCT01695239) and SPIRIT-P2 (NCT02349295) [18, 19] multicenter, double-blind, randomized, placebo-controlled, phase 3 trials evaluating the efficacy and safety of ixekizumab in patients with active PsA. In SPIRIT-P1, patients were randomized 1:1:1:1 to receive subcutaneous injections of placebo (data not reported here), adalimumab 40 mg once every two weeks (Q2W) (data not reported here), ixekizumab 80 mg Q2W (data not reported here), or ixekizumab 80 mg once every four weeks (Q4W) for 24 weeks. After week 24, patients receiving ixekizumab remained on their originally assigned dose, and those receiving placebo or adalimumab were re-randomized 1:1 to receive ixekizumab Q2W or Q4W through week 156. In SPIRIT-P2, patients were randomized 1:1:1 to receive subcutaneous injections of placebo (data not reported here), ixekizumab 80 mg Q2W (data not reported here), or ixekizumab 80 mg Q4W for 24 weeks. In both studies at week 16, patients who failed to meet predefined criteria for change in tender joint count (TJC) and swollen joint count (SJC) from baseline were classified as inadequate responders (< 20% improvement from baseline in TJC and SJC) and were administered rescue therapy through week 24 [16]. Patients who failed to demonstrate a ≥ 20% improvement from baseline in both tender joint and swollen joint counts at week 32 or thereafter were discontinued from the studies. Changes in concomitant medications were not allowed from weeks 0 through 24 with the exception of inadequate responders who were administered rescue therapy or patients who changed medication due to safety reasons; changes were allowed after week 24 through week 156. Additional details on the study designs have been published previously [18, 19].

### Patients

Patients eligible for SPIRIT-P1 and SPIRIT-P2 were 18 years of age or older with an established diagnosis of PsA for at least 6 months, met Classification for Psoriatic Arthritis criteria, had active psoriatic skin lesions or a documented history of plaque psoriasis, and had active PsA as defined by the presence of at least 3/68 tender and 3/66 swollen joints. bDMARD-naïve patients in SPIRIT-P1 were stratified by csDMARD experience into naïve, past-use, and current-use groups. Patients in SPIRIT-P2 were bDMARD-experienced, were previously treated with ≥ 1 csDMARD, and had an inadequate response (≥ 12 weeks on therapy) or intolerance to 1 or 2 tumor necrosis factor (TNF) alpha inhibitors. Patients must have been on a stable dose of a csDMARD for at least 8 weeks prior to baseline and were not permitted to use more than 1 csDMARD upon study entry. In SPIRIT-P1, radiographs were taken of both hands and feet at screening and were reviewed centrally (by 2 primary readers and an adjudicator when necessary) for evidence of erosive bony changes. As part of the inclusion criteria, patients were required to have at least 1 PsA-related joint erosion on hand or foot radiographs or a C-reactive protein (CRP) level of at least 6 mg/L to be enrolled into SPIRIT-P1.

The maximum allowed doses of concomitant csDMARDs were 25 mg/week for MTX, 400 mg/day for hydroxychloroquine, 20 mg/day for leflunomide, and 3 g/day for sulfasalazine. Simultaneous use of MTX and leflunomide was prohibited for safety reasons. During the double-blind treatment period from weeks 0 to 24, modifying the dose of a concomitant csDMARD and/or the introduction of a new csDMARD was not allowed except for safety reasons or rescue therapy. Lowering or stopping doses of csDMARDs during the double-blind treatment period was allowed if the investigator believed any adverse events or laboratory abnormalities could be attributable to the concomitant csDMARD. During the extension and long-term extension periods from weeks 24 to 52 and weeks 52 to 156, respectively, adjustment of csDMARDs (dose change, introduction, or withdrawal) was allowed, though more than one adjustment of csDMARD at a time within a period of 12 weeks was discouraged. Any changes in concomitant csDMARDs administered were recorded. Additional patient-related details, including blinding, randomization, and other eligibility criteria, have been published previously [18, 19].

### Assessments and outcomes

The efficacy and safety of ixekizumab Q4W were evaluated in the following subgroups of patients with active PsA according to the concomitant csDMARD they received: (1) ixekizumab monotherapy (no concomitant MTX or other csDMARDs), (2) ixekizumab and MTX, (3) ixekizumab and any csDMARD (MTX, MTX sodium, sulfasalazine, leflunomide, ciclosporin, hydroxychloroquine, or hydroxychloroquine sulfate), through 156 weeks [20]. Patients in the ixekizumab and MTX subgroup had uninterrupted MTX use (no more than 14 days without using MTX) but were allowed to switch MTX medications and doses. Patients in the ixekizumab and any csDMARD group could have taken concomitant MTX; that is, the ixekizumab and MTX subgroup is a subset of the ixekizumab and any csDMARD subgroup. Categorical outcomes measured include the proportions of patients achieving American College of Rheumatology (ACR) 20/50/70 responses; low disease activity (LDA) as indicated by a score ≤ 14 on the Disease Activity in Psoriatic Arthritis (DAPSA), which is measured by the sum of patient global and pain visual analogue scales (cm), swollen joint count (SJC) of 66 joints, tender joint count (TJC) of 68 joints, and CRP level (mg/dl) [21, 22]; minimal disease activity (MDA), which is achieved if ≥ 5 of the following 7 criteria are met: TJC ≤ 1, SJC ≤ 1, Psoriasis Area and Severity Index (PASI) ≤ 1 or body surface area (BSA) ≤ 3%, patients assessment of pain visual analogue scale (VAS) ≤ 15, patient’s global assessment of disease activity VAS ≤ 20, Health Assessment Questionnaire-Disability Index (HAQ-DI) ≤ 0.5, tender entheseal points ≤ 1; PASI 75/90/100 responses; Nail Psoriasis Severity Index (NAPSI) (0) response; and HAQ-DI improvement from baseline ≥ 0.35 response. Continuous outcomes measured included changes from baseline in NAPSI score and the 36-item Short Form Survey (SF-36) mental and physical functioning domains. For patients in SPIRIT-P1, hand and foot radiographs performed at screening and weeks 52, 108, and 156 were used to evaluate radiographic progression over 3 years. Scoring of radiographs was performed by 2 independent readers, blinded to chronology and clinical data [23]. Structural progression in peripheral joints was measured using the Bone Erosion Score (ES), Joint Space Narrowing (JSN) score, and the van der Heijde modified Total Sharp Score (mTSS), with higher scores indicating greater damage [24]. The initial radiographs obtained at screening served as the baseline radiographs for this analysis.

Safety was evaluated using the incidence of treatment-emergent adverse events (TEAEs) (total, mild, moderate, and severe), serious adverse events (SAEs), adverse events (AEs) leading to discontinuation, and AEs of special interest, which were prespecified. Immunogenicity was evaluated by assessing the number of patients who were positive for treatment-emergent (TE) ADA. Of these patients, neutralizing antibody (Nab) status was also assessed.

### Statistical analyses

The post hoc analyses reported here included all patients who were initially randomized to ixekizumab Q4W treatment. Subgroups were comprised of patients treated with ixekizumab who concomitantly received the following treatments from baseline: (1) ixekizumab monotherapy (no concomitant MTX or csDMARDs); (2) ixekizumab and MTX; or (3) ixekizumab and any csDMARD (including MTX). Categorical variables were reported as percentages, and modified non-responder imputation was used to impute missing data. Continuous variables were reported with multiple imputation (MI) used to impute missing data. Radiographic analyses were conducted in patients enrolled in SPIRIT-P1 who received at least 1 dose of the study drug in the long-term extension period starting at week 24. Linear extrapolation was used to impute missing radiographic progression data if patients had baseline and at least one post-baseline value at week 52, 108, or 156. Cumulative probability plots were presented for radiographic progression through 156 weeks. Safety and immunogenicity were summarized using descriptive statistics. The safety analysis population consisted of all randomized patients who received at least 1 dose of study treatment and who were initially randomized to ixekizumab Q4W at week 0. Statistical analyses were performed using SAS® Version 9.2 or higher.

### Statement of human and animal rights

The SPIRIT-P1 and SPIRIT-P2 clinical trials followed Good Clinical Practice, the Declaration of Helsinki, and local regulations. Approval was given by each additional site. All patients provided written informed consent before participating in the trials.

## Results

### Baseline demographics and disease characteristics

Of 229 patients randomized to ixekizumab Q4W treatment in SPIRIT-P1 and SPIRIT-P2, 202 were categorized into one of three subgroups and included in this integrated post hoc analysis. Of 107 patients initially randomized to ixekizumab Q4W treatment in SPIRIT-P1, 97 completed the double-blind treatment period (weeks 0 to 24), and 63 completed the combined extension and long-term extension period (weeks 24 to 156). Of 122 patients initially randomized to ixekizumab Q4W treatment in SPIRIT-P2, 70 completed the double-blind treatment period, and 70 completed the extension period (weeks 24 to 156). The numbers of patients receiving ixekizumab monotherapy, ixekizumab and MTX, and ixekizumab and any csDMARD (including MTX), comprising the 3 treatment subgroups, were 89, 88, and 113, respectively. In the third subgroup of patients receiving any concomitant csDMARD that includes patients receiving MTX, 24 did not receive MTX continuously. Baseline characteristics were similar across the 3 subgroups (Table 1). Of patients treated with ixekizumab and MTX, the mean MTX dose at baseline was 15.7 mg/week. Concomitant medications in the ixekizumab and any csDMARD subgroup included MTX, sulfasalazine, leflunomide, ciclosporin, hydroxychloroquine, and hydroxychloroquine sulfate.

**Table 1:** Demographics and baseline disease characteristics of patients from SPIRIT-P1 and SPIRIT-P2 treated with ixekizumab Q4W

Concomitant background treatment	Ixekizumab monotherapy^a^ *Ns* = 89	Ixekizumab + MTX *Ns* = 88	Ixekizumab + any csDMARD^b^ *Ns* = 113
Age (years)	51.1 (11.9)	51.0 (12.1)	50.1 (12.4)
Male, *n* (%)	43 (48.3)	41 (46.6)	54 (47.8)
Race, *n* (%)			
White	86 (96.6)	80 (90.9)	103 (91.2)
Asian	1 (1.1)	3 (3.4)	5 (4.4)
American Indian or Alaska Native	0	2 (2.3)	2 (1.8)
Native Hawaiian or other Pacific Islander	0	1 (1.1)	1 (0.9)
Black or African American	1 (1.1)	0	0
Multiple	1 (1.1)	2 (2.3)	2 (1.8)
Weight, kg	87.8 (22.7)	87.4 (20.1)	87.3 (20.3)
BMI, kg/m^2^	30.0 (7.3)	30.6 (6.7)	30.5 (6.8)
Previous PsA systemic therapy, *n* (%)			
No prior treatment	8 (9.0%)	25 (28.4%)	29 (25.7%)
Non-biologic only	28 (31.5%)	23 (26.1%)	30 (26.5%)
TNFi only	0	21 (23.9%)	24 (21.2%)
TNFi and non-biologic	53 (59.6%)	19 (21.6%)	30 (26.5%)
Corticosteroid use, *n* (%)	13 (14.6)	12 (13.6)	14 (12.4)
Time since PsA diagnosis, years	17.5 (13.8)	14.6 (12.8)	14.8 (12.7)
Baseline disease characteristics			
	*n* = 88	*n* = 88	*n* = 113
Tender joints (68 assessed)	22.0 (13.7)	20.7 (14.8)	21.0 (14.4)
	*n* = 88	*n* = 88	*n* = 113
Swollen joints (66 assessed)	12.2 (8.6)	11.6 (10.6)	12.3 (11.2)
	*n* = 89	*n* = 88	*n* = 113
LEI > 0, *n* (%)	49 (55.1)	54 (61.4)	67 (59.3)
	*n* = 87	*n* = 87	*n* = 110
LDI-B > 0, *n* (%)	24 (27.6)	26 (29.9)	35 (31.8)
	*n* = 87	*n* = 82	*n* = 107
PASI	7.4 (8.7)	6.4 (6.3)	6.2 (6.2)
	*n* = 60	*n* = 65	*n* = 78
NAPSI^c^	24.2 (22.7)	17.9 (16.3)	18.7 (16.2)
	*n* = 89	*n* = 83	*n* = 106
% BSA	13.3 (18.4)	14.0 (15.8)	13.6 (15.7)
SF-36	*n* = 87	*n* = 85	*n* = 110
PCS	32.5 (9.6)	32.6 (9.5)	32.7 (9.5)
MCS	47.3 (13.6)	46.4 (12.5)	46.6 (12.3)
Baseline radiographic scores^d^	n1/N1 = 23/36	n1/N1 = 32/48	n1/N1 = 41/59
ES^d^	9.7 (13.6)	10.8 (17.4)	11.0 (17.0)
JSN^d^	6.7 (13.3)	8.4 (19.7)	8.5 (18.4)
mTSS^d^	16.5 (26.8)	19.2 (36.4)	19.5 (34.8)

Values are mean (SD) unless otherwise indicated

All patients were initially randomized to ixekizumab

^a^Patients receiving no MTX or other csDMARDs

^b^Patients receiving any csDMARD, including MTX

^c^Patients initially randomized to ixekizumab with fingernail involvement at baseline

^d^Patients from the SPIRIT-P1 trial

*BSA*, body surface area; *csDMARD*, conventional synthetic disease-modifying antirheumatic drug; *ES*, Bone Erosion Score; *IXE*, ixekizumab; *JSN,* Joint Space Narrowing score; *LDI-B*, Leeds Dactylitis Index-Basic; *LEI*, Leeds Enthesitis Index; *MCS*; mental component score; *mTSS*, modified Total Sharp Score; *MTX*, methotrexate; *Ns*, number of patients in the treatment subgroup; *N1*, number of patients in the specified treatment subgroup from SPIRIT-P1; *n*, number of patients in the specified category; *n1*, number of patients in the specified category from SPIRIT-P1; *NAPSI*, Nail Psoriasis Severity Index; *PCS*, physical component score; *PASI*, Psoriasis Area and Severity Index; *Q4W*, every 4 weeks; *SD*, standard deviation; *SF-36*, 36-Item Short Form Health Survey; *TNFi,* tumor necrosis factor inhibitor

### Responses and changes from baseline in composite measures

**Fig. 1:**
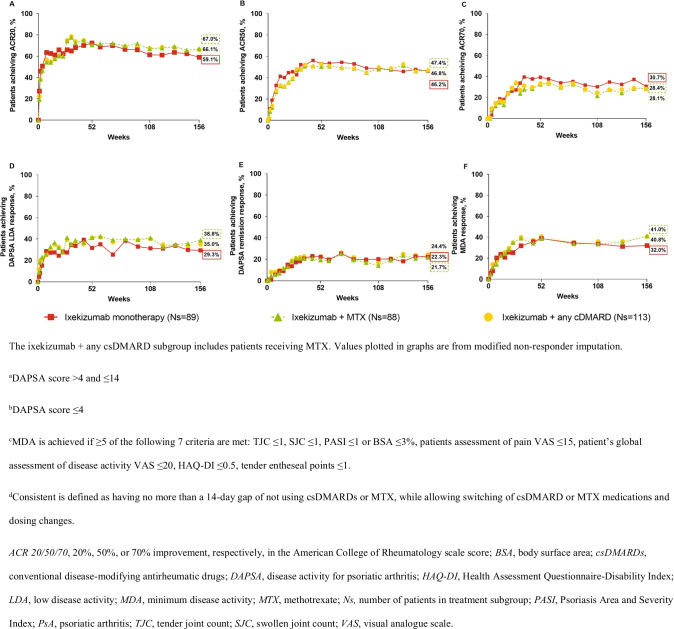
Clinical response and disease control. **A** ACR20, **B** ACR50, **C** ACR70, **D** DAPSA LDA^a^, **E** DAPSA remission^b^, and **F** MDA^c^ % response in patients with PsA and treatment with ixekizumab Q4W and either ixekizumab monotherapy or consistent^d^ concomitant MTX or any csDMARD (including MTX) through three years (156 weeks)

**Fig. 2:**
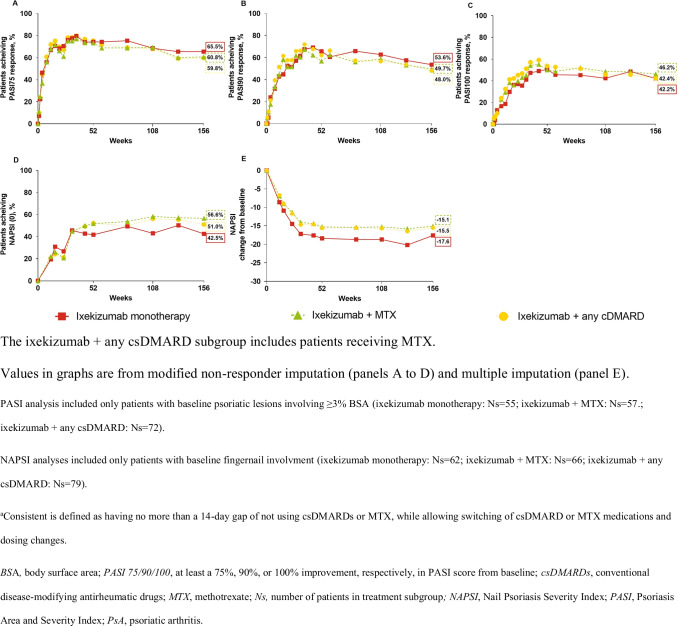
Psoriasis skin lesions and nail involvement. **A** PASI 75, **B** PASI 90, **C** PASI 100, **D** NAPSI (0) % response, and **E** NAPSI mean change from baseline in patients with PsA and treatment with ixekizumab Q4W and either ixekizumab monotherapy or consistent^a^ concomitant MTX or any csDMARD (including MTX) through three years (156 weeks)

Improvement in signs and symptoms of PsA in patients treated with ixekizumab was observed through week 156 regardless of whether ixekizumab was used as monotherapy or with concomitant MTX or other csDMARDs as assessed by ACR 20/50/70 responses (Fig. 1A–C). Similar results were observed for DAPSA LDA and remission responses and MDA responses (Fig. 1D–F). Psoriasis severity, as measured by PASI 75/90/100 responses, and fingernail involvement, as measured by NAPSI (0) response and NAPSI change from baseline, improved through week 156 whether ixekizumab was used as monotherapy or with concomitant MTX or other csDMARDs (Fig. 2A–D). With respect to the quality of life as measured by changes from baseline (MI analysis) in SF-36 PCS and MCS and in functional disability as measured by improvement from baseline of at least 0.35 in HAQ-DI, benefits were observed whether ixekizumab was used as monotherapy or with MTX or other csDMARDs through week 156 regardless of concomitant csDMARD use (Suppl. Fig. 1A–C).

### Radiographic progression

Radiographic progression of structural joint damage was assessed in the SPIRIT-P1 trial only. Changes from baseline in ES, JSN, and mTSS were similar across the three subgroups through 156 weeks with several notable outliers who had significant damage at baseline (Suppl. Table 2).

**Fig. 3:**
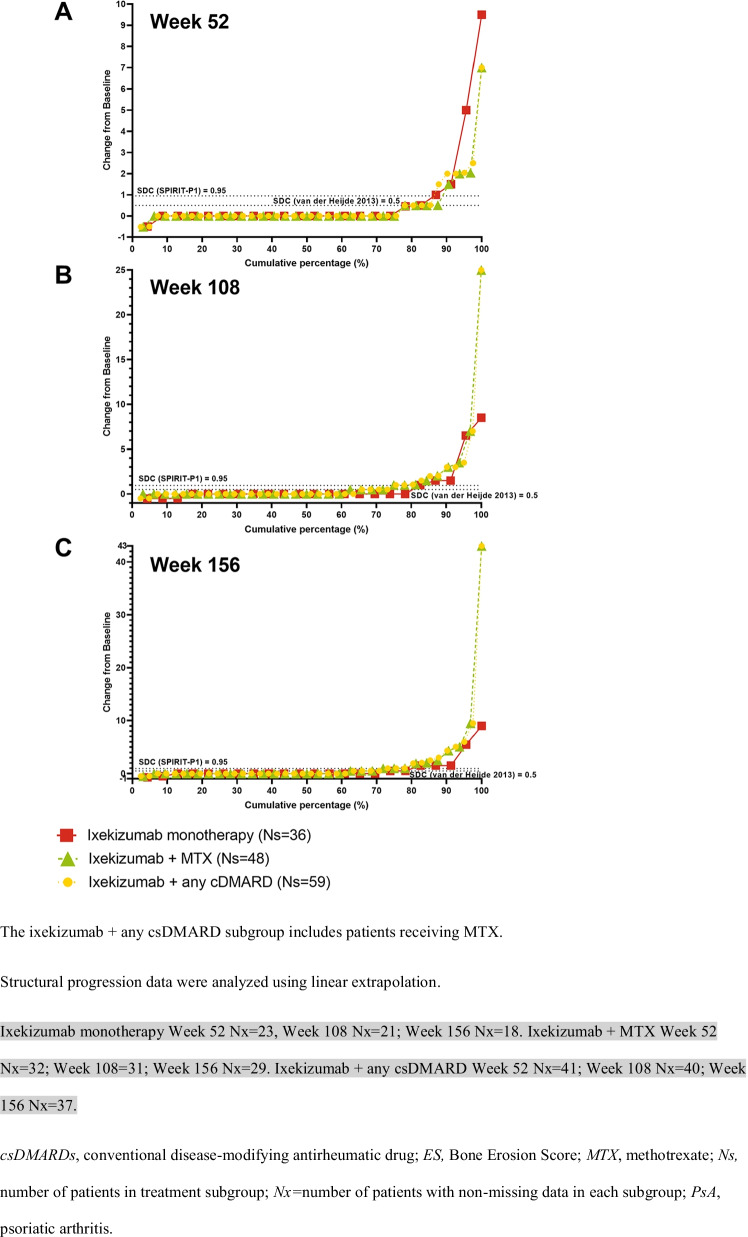
Cumulative probability of change from baseline in structural joint damage as measured by ES in patients from SPIRIT-P1 with PsA and treatment with ixekizumab Q4W as monotherapy or with concomitant MTX or any csDMARD (including MTX) at **A** 52, **B** 108, and **C** 156 weeks

**Fig. 4:**
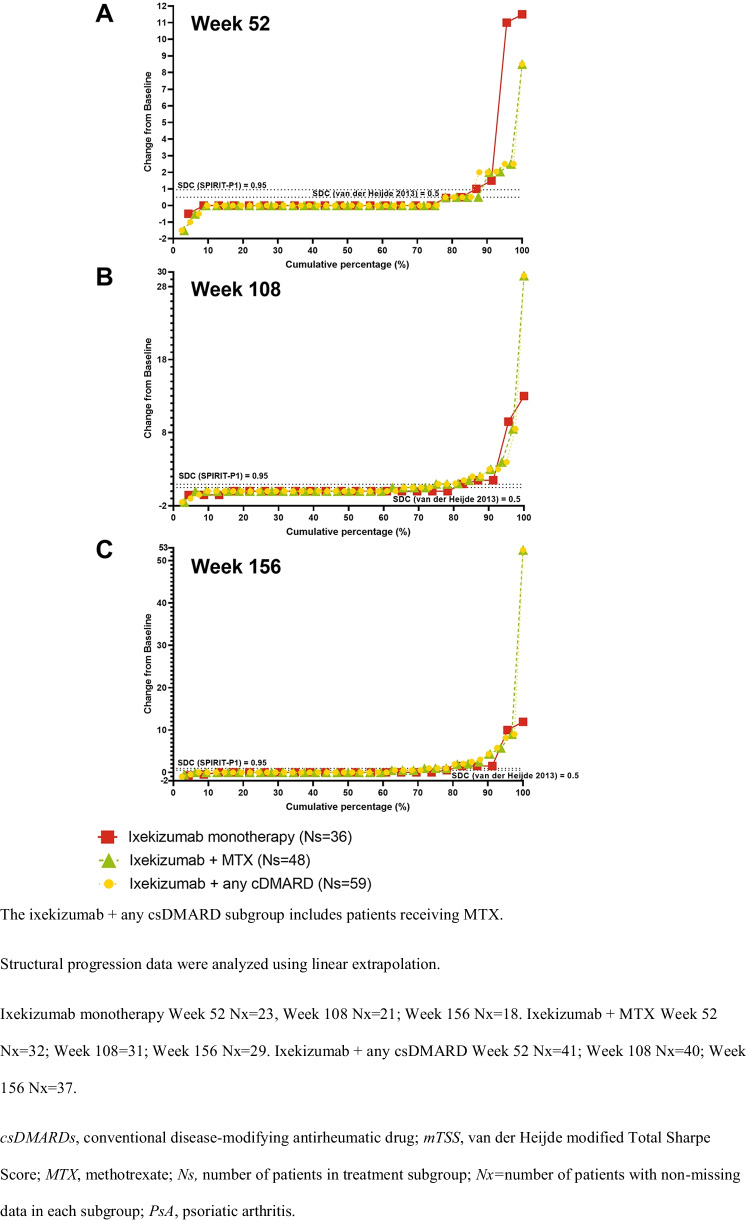
Cumulative probability of change from baseline in structural joint damage as measured by mTSS in patients from SPIRIT-P1 with PsA and treatment with ixekizumab Q4W as monotherapy or with concomitant MTX or any csDMARD (including MTX) at **A** 52, **B** 108, and **C** 156 weeks

Figure 3, Supplemental Fig. 3, and Fig. 4 show cumulative probability plots for changes in baseline in Bone ES, JSN, and mTSS, which illustrate the impact of the outlier scores of several patients in the ixekizumab and MTX (only) and ixekizumab and csDMARDs (any) subgrouTwo patients receiving ixekizumab monotherapy and one patient receiving ixekizumab and MTX (all enrolled in SPIRIT-P1) experienced continued worsening of ES, JSN, and mTSS scores at 1 year and through 3 years despite treatment. All three patients shared higher TJC and SJC counts at baseline, and one patient had comorbid osteopenia. Mean baseline TJC and SJC for the 3 treatment subgroups ranged from 20.7 to 22.0 and 11.6 to 12.3, respectively, and mean baseline ES, JSN, and mean mTSS scores ranged from 9.7 to 11.0, 6.7 to 8.5, and 16.5 to 19.5, respectively (Table 1). In comparison, the baseline TJC and SJC scores for the patients who were outliers ranged from 25 to 32 and 12 to 33, respectively. The baseline ES, JSN, and mTSS scores for the patients who were outliers ranged from 27.5 to 53, 22 to 46.4, and 49.5 to 99.9, respectively.

### Safety

Most TEAEs were mild or moderate in severity (Table 2). Similar proportions of patients experienced at least one TEAE across all ixekizumab treatment subgroups, though incidence rates (IRs) of moderate TEAEs were numerically higher for patients with ixekizumab monotherapy than those with ixekizumab and concomitant MTX or any csDMARD, Incidence rates (IRs) of SAEs were also similar across the subgroups. Rates of discontinuation due to AEs were numerically higher for patients receiving ixekizumab monotherapy compared to those receiving ixekizumab and MTX or ixekizumab and any csDMARD (Table 2). IRs of infections were numerically higher for patients receiving ixekizumab monotherapy compared to the other two subgroups; however, IRs of serious infections were similar across the three subgroups. Injection site reactions were also numerically higher for patients receiving ixekizumab monotherapy compared to the other subgroups. Through three years, IRs of infections and injection site reactions in patients receiving ixekizumab monotherapy decreased year by year, and most of these adverse events were mild in severity.

**Table 2:** Safety overview after 156 weeks of treatment with ixekizumab Q4W according to concomitant csDMARD or MTX use (incidence rates per 100 PY)

Parameter	Ixekizumab monotherapy^a^ *Ns* = 89		Ixekizumab + MTX *Ns* = 88		Ixekizumab + any csDMARD^b^ *Ns* = 113	
Total PY	188.3		201.1		256.0	
	*n* (%)	IR	*n* (%)	IR	*n* (%)	IR
TEAEs (≥ 1)	81 (91.0)	43.0	74 (84.1)	36.8	94 (83.2)	36.7
Mild	25 (28.1)	13.3	27 (30.7)	13.4	33 (29.2)	12.9
Moderate	49 (55.1)	26.0	38 (43.2)	18.9	51 (45.1)	19.9
Severe	7 (7.9)	3.7	9 (10.2)	4.5	10 (8.8)	3.9
SAEs	13 (14.6)	6.9	12 (13.6)	6.0	13 (11.5)	5.1
Discontinuations due to AE	11 (12.4)	5.8	6 (6.8)	3.0	11 (9.7)	4.3
AEs of special interest	72 (80.9)	38.2	64 (72.7)	31.8	81 (71.7)	31.6
Infections	67 (75.3)	35.6	47 (53.4)	23.4	61 (54.0)	23.8
Nasopharyngitis	22 (24.7)	11.7	8 (9.1)	4.0	11 (9.7)	4.3
Upper respiratory tract infection	18 (20.2)	9.6	12 (13.6)	6.0	17 (15.0)	6.6
Sinusitis	8 (9.0)	4.2	7 (8.0)	3.5	8 (7.1)	3.1
Bronchitis	7 (7.9)	3.7	5 (5.7)	2.5	9 (8.0)	3.5
Serious infections	3 (3.4)	1.6	3 (3.4)	1.5	3 (2.7)	1.2
Serious *Candida* infection	1 (1.1)	0.5	–	–	–	–
Serious latent tuberculosis	1 (1.1)	0.5	–	–	–	–
Serious pneumonia	1 (1.1)	0.5	2 (2.3)	1.0	2 (1.8)	0.8
Serious gastroenteritis	–	–	1 (1.1)	0.5	1 (0.9)	0.4
Injection-site reactions	19 (21.3)	10.1	11 (12.5)	5.5	16 (14.2)	6.3
Hepatic events	8 (9.0)	4.2	6 (6.8)	3.0	8 (7.1)	3.1
Allergic reactions/hypersensitivities	7 (7.9)	3.7	4 (4.5)	2.0	8 (7.1)	3.1
Non-anaphylaxis	7 (7.9)	3.7	4 (4.5)	2.0	8 (7.1)	3.1
Depression	3 (3.4)	1.6	5 (5.7)	2.5	6 (5.3)	2.3
Malignancies	3 (3.4)	1.6	1 (1.1)	0.5	2 (1.8)	0.8
Cerebrocardiovascular events	1 (1.1)	0.5	1 (1.1)	0.5	1 (0.9)	0.4
Inflammatory bowel disease	–	–	–	–	–	–

^a^Patients receiving no MTX or other csDMARDs

^b^Patients receiving any csDMARD, including MTX

*AE*, adverse event; *csDMARDs*, conventional disease-modifying anti-rheumatic drugs; *CI*, confidence interval; *IXE*, ixekizumab; *IR*, incidence rate; *PY*, patient years; *Q4W*, every 4 weeks; *SAE*, serious adverse event; *MTX*, methotrexate; *Ns*, number of patients in treatment subgroup; *n*, number of patients in specified category; *TEAE*, treatment-emergent adverse event

### Immunogenicity

A numerically greater proportion of patients receiving ixekizumab monotherapy (15.9%) were TE-ADA positive compared to those receiving ixekizumab and MTX (13.1%) or ixekizumab and any csDMARD (11.0%) (Supplemental Table 1). Of patients who were TE-ADA positive, most had low titer status (92.9%, 90.9%, and 91.7% in the ixekizumab monotherapy, ixekizumab and MTX, and ixekizumab and any csDMARD subgroups, respectively). Of patients who were TE-ADA positive, 35.7%, 27.3%, and 25.0%, in the three treatment subgroups, respectively, had positive Nab status.

## Discussion

The analyses reported here show that ixekizumab improves signs and symptoms of PsA, including manifestations of psoriasis, and quality of life in patients with active PsA up to 156 weeks, whether used as monotherapy or with concomitant MTX or other csDMARDs. These results confirm and extend previous 24- and 52-week analyses [9, 15, 16], showing consistent long-term efficacy of ixekizumab with or without concomitant therapy with MTX or other csDMARDs.

We assessed the radiographic progression of structural joint damage by the mean change from baseline to weeks 52, 108, and 156 in ES, JSN, and mTSS. For the majority of patients (~ 85%), the changes from baseline were similar across the three treatment groups through 156 weeks (Fig. 3, Suppl. Fig. 2, and Fig. 4). It is important to keep in mind that there were outliers who could have influenced the scores. Three patients receiving ixekizumab monotherapy had outlying ES, JSN, and mTSS scores at 156 weeks compared to the mean; these patients also had higher-than-average ES, JSN, and mTSS scores (all 3 with baseline mTSS scores > 40) as well as TJC and SJC at baseline. Previously described low rates of radiographic progression persisted with up to three years of ixekizumab treatment regardless of the addition of background MTX or csDMARD [23].

The overall safety profile presented here is consistent with previously published ixekizumab safety analyses in patients with PsA [18, 19]. All three ixekizumab treatment subgroups had similar safety findings. Similar frequencies of patients in the three subgroups had at least one treatment-emergent adverse event. Even though this post hoc analysis was not powered to evaluate differences in safety between the groups, numerical differences in frequencies of infections and injection site reactions were present, with patients receiving ixekizumab monotherapy reporting higher rates of these adverse events than those receiving ixekizumab and MTX (only) or ixekizumab and csDMARD (any). Because the concomitant treatment subgroups were not randomized, these differences in frequencies of infections and injection site reactions could be due to bias as those at high risk of infections may stop csDMARD use. Additionally, larger studies may be needed to evaluate the frequencies of these TEAEs in these subgroups of patients.

Immunogenicity against biologics is generally understood to be mitigated by treatment with concomitant immunosuppressants, such as MTX and other csDMARDs [25]; however, concomitant MTX for the sole purpose of preventing or lessening immunogenicity is not currently recommended in psoriasis [26]. While patients receiving ixekizumab monotherapy did have numerically higher ADA compared to those receiving ixekizumab and MTX and ixekizumab and any csDMARD, these differences were very small and not thought to be of clinical consequence. Previous 52-week results from SPIRIT-H2H, a study of ixekizumab versus adalimumab, are consistent with the findings from the present 156-week analysis of bDMARD-naïve and TNFi-experienced patients with PsA enrolled in SPIRIT-P1 and SPIRIT-P2, showing that treatment with ixekizumab demonstrated consistent efficacy with and without concomitant csDMARDs, including MTX [17].

This post hoc analysis was limited as it used RCT data and did not address data from patients in the real world. Because this analysis evaluated efficacy and safety through 3 years, it is potentially biased towards data from patients who remained in the study long term. Subgroup sample sizes were also small. In addition, there were no placebo arms during the long-term extension periods of SPIRIT-P1 and SPIRIT-P2. Radiographic progression of structural joint damage was only assessed in SPIRIT-P1 (enrolling bDMARD-naïve patients) and not in SPIRIT-P2 (enrolling TNFi-experienced patients), restricting the applicability of these results to bDMARD-naïve patients only. In addition, radiographic progression of structural joint damage was only assessed to week 24, limiting our ability to confirm the week 52 results from this study. Safety results were limited by small numbers of patients in the treatment groups, which could have impacted the mean scores for the safety assessments. Safety results were also limited by the possibility of bias by indication, as those at high risk of infections may stop csDMARD use.

This analysis’s strengths include a long treatment duration through 156 weeks as well as the inclusion of data from two clinical trials evaluating both bDMARD-naïve and TNFi-experienced patient populations (with the exception of the radiographic progression analyses, which were only performed on data from bDMARD-naïve patients). In addition, this analysis evaluated immunogenicity, including detecting the presence or absence of Nab, to ixekizumab treatment with or without concomitant MTX or csDMARDs in the post-baseline period through 156 weeks.

In conclusion, this post hoc analysis supports that ixekizumab can offer similar efficacy and safety profiles whether it is used as monotherapy or as combination therapy with MTX or other csDMARDs. More dedicated studies are needed to understand the radiographic progression findings. The safety profile for ixekizumab is similar to what has been previously reported with no new safety signals. The proportions of patients who had ADAs and Nab in the post-baseline period through 156 weeks were small and similar in patients receiving ixekizumab with or without concomitant MTX or csDMARDs. These data suggest that there are no additional clinical or radiographic benefits to concomitant MTX or other csDMARD use in this post hoc analysis, supporting the use of ixekizumab monotherapy.

## Supplementary Information

ESM 1 (DOCX 531 kb)

## Data Availability

Lilly provides access to all individual participant data collected during the trial, after anonymization, with the exception of pharmacokinetic or genetic data. Data are available to request 6 months after the indication studied has been approved in the US and EU and after primary publication acceptance, whichever is later. No expiration date of data requests is currently set once data are made available. Access is provided after a proposal has been approved by an independent review committee identified for this purpose and after receipt of a signed data sharing agreement. Data and documents, including the study protocol, statistical analysis plan, clinical study report, blank, or annotated case report forms, will be provided in a secure data sharing environment. For details on submitting a request, see the instructions provided at www.vivli.org.
