# Correction to: Efficacy and safety of ixekizumab in patients with active psoriatic arthritis with and without concomitant conventional disease-modifying antirheumatic drugs: SPIRIT-P1 and SPIRIT-P2 3-year results

**DOI:** 10.1007/s10067-022-06271-3

**Published:** 2022-07-11

**Authors:** Laura C. Coates, Philip Mease, Andris Kronbergs, Cameron Helt, David Sandoval, So Young Park, Bernard Combe, Peter Nash, Atul Deodhar

**Affiliations:** 1 Nuffield Department of Orthopaedics, Rheumatology and Musculoskeletal Sciences, University of Oxford, Oxford, UK; 2 Swedish Medical Center/Providence St, Joseph Health and University of Washington, Seattle, WA USA; 3 Eli Lilly and Company, Indianapolis, IN USA; 4 University of Montpellier, Montpellier, France; 5 Department of Medicine, University of Queensland, Queensland, Brisbane Australia; 6 Division of Arthritis and Rheumatic Diseases, Oregon Health & Science University, 3181 S.W. Sam Jackson Park Rd, Portland, OR 97239 USA

**Correction to: Clinical Rheumatology**

**https://doi.org/10.1007/s10067-022-06218-8**

The article “Efficacy and safety of ixekizumab in patients with active psoriatic arthritis with and without concomitant conventional disease-modifying antirheumatic drugs: SPIRIT-P1 and SPIRIT-P2 3-year results,” written by Laura C. Coates, Philip Mease, Andris Kronbergs, Cameron Helt, David Sandoval, So Young Park, Bernard Combe, Peter Nash, and Atul Deodhar, was originally published electronically on the publisher’s internet portal on 16 May 2019 without open access. With the author(s)’ decision to opt for Open Choice, the copyright of the article changed on 08 June 2022 to © The Author(s) 2022 and the article is forthwith distributed under a Creative Commons Attribution 4.0 International License, which permits use, sharing, adaptation, distribution, and reproduction in any medium or format, as long as you give appropriate credit to the original author(s) and the source, provide a link to the Creative Commons licence, and indicate if changes were made. The images or other third-party material in this article are included in the article’s Creative Commons licence, unless indicated otherwise in a credit line to the material. If material is not included in the article’s Creative Commons licence and your intended use is not permitted by statutory regulation or exceeds the permitted use, you will need to obtain permission directly from the copyright holder. To view a copy of this licence, visit http://creativecommons.org/licenses/by/4.0.

