# Supplementary material for: Efficacy and safety of ixekizumab in patients with active psoriatic arthritis with and without concomitant conventional disease-modifying antirheumatic drugs: SPIRIT-P1 and SPIRIT-P2 3-year results
Source: Clin Rheumatol. 2022 Jun 8;41(10):3035–47. doi: 10.1007/s10067-022-06218-8 (PMC9485169; doi:10.1007/s10067-022-06218-8)
Supplement: Supplementary file 1 — (DOCX 531 kb) [file 10067_2022_6218_MOESM1_ESM.docx]

**Supplemental Material**

Supplemental Table 1 Immunogenicity by treatment-emergent anti-drug antibody status through 156 weeks of treatment with ixekizumab Q4W according to concomitant cDMARD or MTX use

|  | Ixekizumab monotherapy^a^ Ns=89 | Ixekizumab + MTX  Ns=88 | Ixekizumab + any cDMARD^b^ Ns=113 |
| --- | --- | --- | --- |
| Post-baseline period (through Week 156) | | | |
| TE-ADA negative, n (%)^c^ | 74 (84.1) | 73 (86.9) | 97 (89.0) |
| TE-ADA positive, n (%)^c^ | 14 (15.9) | 11 (13.1) | 12 (11.0) |
| TE-ADA positive titer status, n (%)^d^ |  |  |  |
| Low (<1:160) | 13 (92.9) | 10 (90.9) | 11 (91.7) |
| Moderate (≥1:160 and <1:1280) | 1 (7.1) | 0 | 0 |
| High (≥1:1280) | 0 | 1 (9.1) | 1 (8.3) |
| TE-ADA positive Nab status, n (%)^d^ |  |  |  |
| Positive^e^ | 5 (35.7) | 3 (27.3) | 3 (25.0) |
| Negative^f^ | 2 (14.3) | 1 (9.1) | 1 (8.3) |
| Inconclusive^g^ | 7 (50.0) | 7 (63.6) | 8 (66.7) |

^a^Patients receiving no MTX or other cDMARDs. ^b^Patients receiving any cDMARD, including MTX. ^c^Percentage based on the number of ADA-evaluable patients. ^d^Percentage based on number of ADA-evaluable patients who are ADA-positive for baseline, TE-ADA positive for the postbaseline period, and TE-ADA positive at least once for the time-varying TE-ADA titer status. ^e^NAb-positive result for ≥1 TE-ADA positive sample. ^f^Patients who were NAb evaluable and were not NAb-positive or NAb-inconclusive, ^g^Patients without a NAb-positive sample and with ≥1 sample for which drug levels may have interfered with the NAb assay.

*ADA=*anti-drug antibody; MTX=methotrexate; NAb=neutralizing antibody; *Ns,* number of patients in treatment subgroup; Nx=number of ADA-evaluable patients; TE-ADA=treatment-emergent anti-drug antibody

Supplemental Table 2 Mean changes from baseline in Bone ES, mTSS, and JSN in patients from SPIRIT-P1 with PsA and treatment with ixekizumab once every 4 weeks as monotherapy or with concomitant MTX or any cDMARD (including MTX) at Weeks 52, 108, and 156

|  | Ixekizumab monotherapy^a^ Ns=36 | Ixekizumab + MTX  Ns=48 | Ixekizumab + any cDMARD^b^Ns=59 |
| --- | --- | --- | --- |
| Week 52 | | | |
| Bone ES | 0.76 (2.20) | 0.44 (1.33) | 0.44 (1.26) |
| JSN | 0.35 (1.38) | 0.03 (0.40) | 0.01 (0.36) |
| mTSS | 1.11 (3.23) | 0.47 (1.64) | 0.45 (1.53) |
| Week 108 | | | |
| Bone ES | 0.76 (2.21) | 1.47 (4.53) | 1.25 (4.05) |
| JSN | 0.33 (1.36) | 0.16 (0.88) | 0.11 (0.79) |
| mTSS | 1.09 (3.29) | 1.63 (5.38) | 1.36 (4.79) |
| Week 156 | | | |
| Bone ES | 0.82 (2.17) | 2.26 (7.70) | 1.98 (6.87) |
| JSN | 0.30 (1.37) | 0.30 (1.69) | 0.28 (1.52) |
| mTSS | 1.12 (3.17) | 2.56 (9.33) | 2.26 (8.33) |

The ixekizumab + any cDMARD subgroup includes patients receiving MTX. Values are mean (SD). Structural progression data were analyzed using the linear extrapolation method.

Ixekizumab monotherapy Week 52 Nx=23, Week 108 Nx=21; Week 156 Nx=18. Ixekizumab + MTX Week 52 Nx=32; Week 108=31; Week 156 Nx=29. Ixekizumab + any cDMARD Week 52 Nx=41; Week 108 Nx=40; Week 156 Nx=37.

*cDMARDs*, conventional disease-modifying antirheumatic drug; *ES*, Bone Erosion Score; *JSN,* Joint Space Narrowing score; *mTSS,* modified Total Sharp Score; *MTX*, methotrexate; *Ns*, number of patients in treatment subgroup; *Nx=*number of patients with non-missing data in each subgroup; PsA, psoriatic arthritis; *SD,* standard deviation.


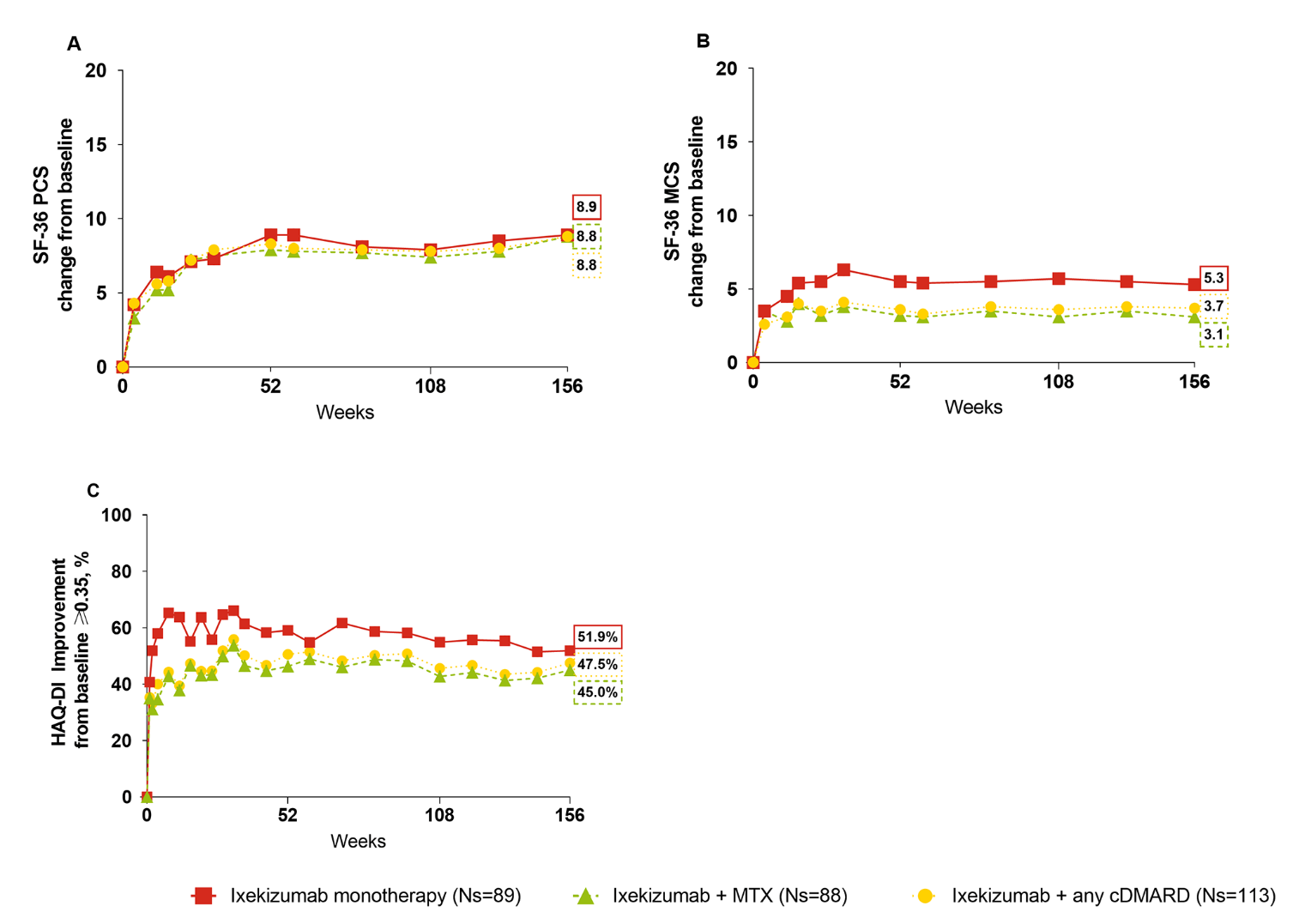


Supplemental Figure 1 Quality of life outcomes. (A) SF-36 PCS change from baseline (CFB), (B) SF-36 MCS CFB, (C) HAQ-DI improvement from baseline ≥0.35 % response in patients with PsA and treatment with ixekizumab once every 4 weeks and either ixekizumab monotherapy or consistent^a^ concomitant MTX or any cDMARD (including MTX) through 3 years (156 weeks)

The ixekizumab + any cDMARD subgroup includes patients receiving MTX.

Values in graphs are from multiple imputation (panels A and B) and modified nonresponder imputation (panel C) analyses.

HAQ-DI analysis included only patients with baseline HAQ-DI ≥0.35

^a^Consistent is defined as having no more than a 14-day gap of not using cDMARDs or MTX, while allowing switching of cDMARD or MTX medications and dosing changes.

*cDMARDs*, conventional disease-modifying antirheumatic drugs; *HAQ-DI*, Health Assessment Questionnaire-Disability Index; *MCS*, mental component score *MTX*, methotrexate; *MI*, multiple imputation; *Ns,* number of patients in treatment subgroup; *PCS*, physical component score; PsA, psoriatic arthritis; *SF-36*, 36-Item Short Form Health Survey


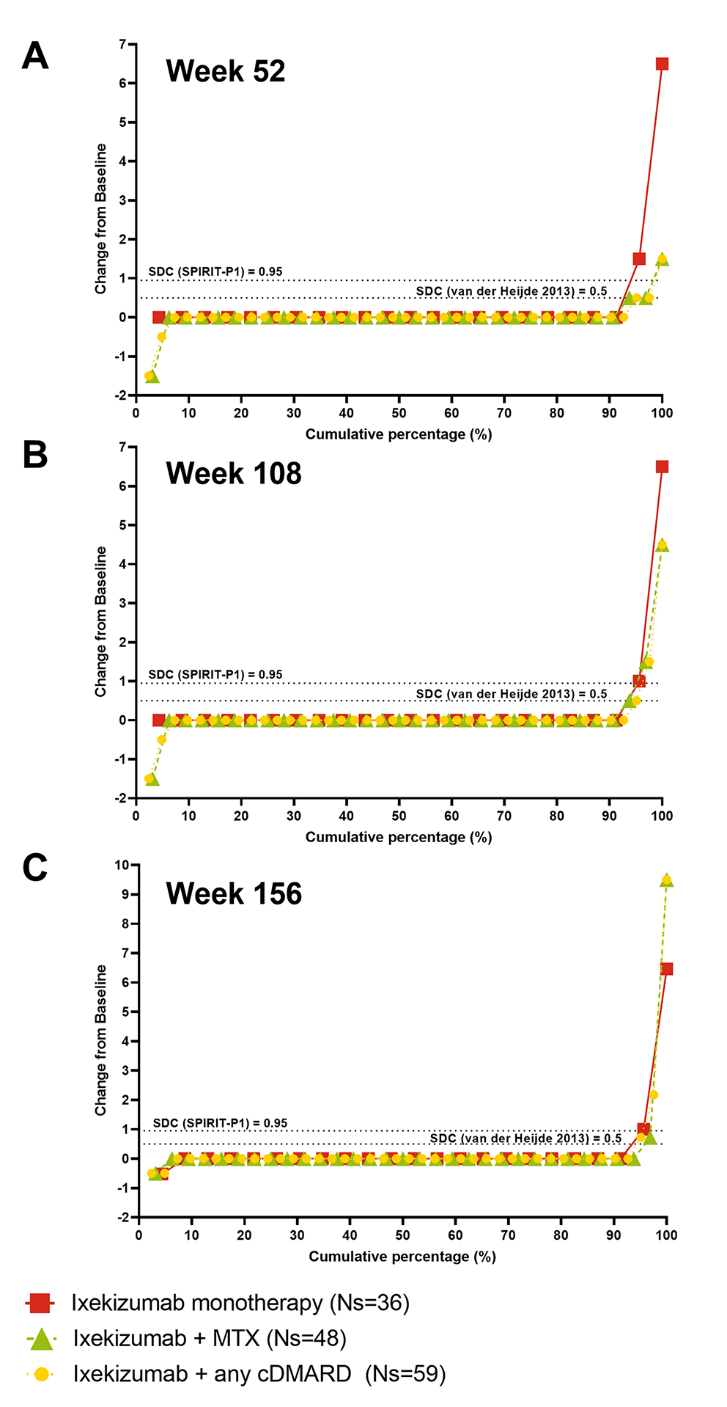


Supplemental Figure 2 Cumulative probability of change from baseline in structural joint damage as measured by JSN in patients from SPIRIT-P1 with PsA and treatment with ixekizumab once every 4 weeks as monotherapy or with concomitant MTX or with any cDMARD (including MTX) at A) 52, B) 108, and C) 156 weeks

The ixekizumab + any cDMARD subgroup includes patients receiving MTX.

Structural progression data were analyzed using linear extrapolation.

Ixekizumab monotherapy Week 52 Nx=23, Week 108 Nx=21; Week 156 Nx=18. Ixekizumab + MTX Week 52 Nx=32; Week 108=31; Week 156 Nx=29. Ixekizumab + any cDMARD Week 52 Nx=41; Week 108 Nx=40; Week 156 Nx=37.

*cDMARDs*, conventional disease-modifying antirheumatic drug; *JSN,* Joint Space Narrowing; *Ns,* number of patients in treatment subgroup; *Nx=*number of patients with non-missing data in each subgroup; *MTX*, methotrexate; PsA, psoriatic arthritis
